# Special Issue: State of the Art of Cardiac Multimodality Imaging

**DOI:** 10.3390/jcm11133793

**Published:** 2022-06-30

**Authors:** Arturo Evangelista, Jose Rodríguez-Palomares

**Affiliations:** Servei de Cardiologia, University Hospital Vall d’Hebron, 08035 Barcelona, Spain; jfrodriguezpalomares@gmail.com

Imaging has progressed significantly in recent years and plays a pivotal role in the diagnosis, prognosis, and management of cardiac diseases. Most imaging techniques offer different information about the disease and the integration of their results may significantly improve the diagnosis and status definition of cardiac diseases. Echocardiography (ECHO) is the most commonly used technique in clinical practice due to its availability, rapidity, and ability to provide adequate anatomical and dynamic information about blood flow by the Doppler technique. Computed tomography (CT) is the imaging technique with the best spatial resolution, giving the most accurate anatomical information about the heart, aorta, and peripheral vessels. Cardiac magnetic resonance imaging (CMR) by different sequences may add morphological and dynamic information; avoid radiation; and offer tissue characterization, which is crucial in several cardiac diseases. However, with this large armamentarium of multimodality imaging, it is important to avoid redundant and duplicated information. Each test must be selected in an integrated and rational way in order to provide clear answers to specific clinical questions, taking into account test accuracy, the benefits of additional information, risks, and cost. In this Special Issue, we examine recent innovations and advances in multimodality imaging for improving the knowledge and management of several cardiac diseases.

Mitral valve prolapse is the most common valve disease, occurring in 3% of the general population. Its prevalence is low among children and young adults, suggesting that it is a progressive degenerative disease. Mitral valve prolapse is a benign disease, associated with non-specific symptoms. The complications associated with this valve disease include mitral regurgitation, infective endocarditis, and cerebrovascular ischemic events- Exceptionally, a subset of patients can experience cardiac arrest or sudden cardiac death due to complex ventricular arrhythmias with a specific phenotype, including thickened leaflets, fibrosis of papillary muscles and the inferobasal wall, and mitral annulus disjunction. This annulus disjunction is defined as an abnormal insertion of the hinge line of the posterior mitral leaflet on the atrial wall—in order words, an atrialization of the posterior leaflet base. Alenazy, A. et al. [1] describes, in an excellent review, the recent advances of multimodality imaging in the assessment of morphologic characteristics of mitral valvular prolapse and the role of CMR in the evaluation and risk stratification of this disease. Furthermore, multimodality imaging can guide treatment, allowing the identification of different, mitral valve prolapse phenotypes.

Bicuspid aortic valve (BAV) is the most common congenital heart disease, with a reported prevalence of 0.8–1.5%. In this issue, Evangelista, A. et al. [2] reports on the consensus regarding BAV nomenclator and the new evidence obtained from the multimodality imaging of BAV and aorta dilation. Familial screening by ECHO identified the presence of BAV in 5–11% of first-degree relatives. Moreover, the frequency of aorta dilation is nearly 10% in those with tricuspid aortic valves. A recent publication reported the presence of a mini-raphe by CT in 41% of first-degree relatives with apparent tricuspid aortic valve and aorta dilation by ECHO.

4D-flowCMR is very useful in the evaluation of abnormal ascending aorta flow in BAV patients. Several studies have shown the flow in the aorta of BAV patients to be eccentric. Such flow abnormalities produce changes in wall shear stress (WSS), which is the force per unit of area exerted tangentially by blood on the aortic wall, which is associated with elastic fiber degeneration and extracellular matrix dysregulation. Higher shear stress values were found at the more proximal tract of the aorta with BAV-RL. However, the RN morphotype spared dilation at the root level and showed higher in-plane shear stress in distal ascending aorta. In a longitudinal study, in patients with BAV, the circumferential wall shear stress component was found to independently predict the progressive dilation of the ascending aorta. Notably, the mapping of WSS further allowed for establishing ascending aorta regions with the fastest dilation rates.

López, A., et al. [3], reports the results of a retrospective study of 718 consecutive patients with BAV followed for more than 5 years. Multivariate analysis showed basal valvular dysfunction severity, hypertension, dyslipidemia, the presence of raphe, and follow-up period to be associated with aortic stenosis or aortic regurgitation progression. Valve calcification progression was found to be greater in patients with hypertension, diabetes and dyslipidemia and in those with raphe. In addition, the annual ascending aorta growth rate was 0.43 mm/year, being greater in patients with hypertension and in those with significant aortic stenosis. Aortic root diameters progressed a mean of 0.23 mm/year and greater root dilation was found to be significantly associated with male sex, arterial hypertension, smoking, the presence of raphe, BAV-RL morphotype, and aortic regurgitation.

Maximum aorta diameters have important clinical value in the diagnosis, follow-up, and surgical indication of many aortic diseases. However, there is no uniformity among experts regarding aorta diameter quantification by ECHO. Servato et al. [4] compares the aortic root diameter measured by the diastolic leading edge and the systolic inner edge conventions in adult and pediatric patients with inherited cardiovascular diseases. The comparison of the diameters measured by the two conventions in the overall population showed a non-significant underestimation of the diameter measured by the systolic inner edge convention at a root level of 0.28 mm and at a tubular ascending aorta level of 0.17 mm. Therefore, the maximum aorta diameter measured by the leading edge convention in end-diastole and the inner edge convention in mid-systole have similar values, permitting them to be interchangeable when used in clinical practice.

Multimodality cardiac imaging techniques are the cornerstone of cardiomyopathy diagnosis; ECHO should be the first-line imaging modality, but diagnosis should be confirmed by CMR, which will provide more accurate morphologic and functional information, as well as extensive tissue characterization. Casas, G. et al. [5] reports that left ventricular ejection fraction (LVEF) and late gadolinium enhancement (LGE) are two of the main variables used for risk stratification. The quantification of LGE, usually as the percentage of total LV mass, has emerged as a powerful predictor of sudden death in hypertrophic cardiomyopathy. An LGE >15% has been proposed as a risk marker for sudden death. Contrarily, the outcomes of patients with a low amount of LGE (<5%) are comparable to those without LGE. Owing to the strong evidence, “extensive” LGE has been incorporated as a parameter for prophylactic cardioverter-defibrillator implantation in the recent American guidelines, which also recommend performing a CMR every 3–5 years to evaluate disease. LVEF remains the strongest predictor of events in dilated cardiomyopathy; thus, patients with an LVEF ≤35% must be considered for optimal medical treatment and prophylactic defibrillator (with/without cardiac resynchronization therapy). However, additional predictors are required. CMR should be performed at least once in all dilated cardiomyopathy patients since it is the gold-standard technique for the assessment of biventricular volumes, systolic function, and tissue characterization. LGE is common in dilated cardiomyopathy and implies myocardial fibrosis. In left ventricular noncompaction, preserved LVEF and negative LGE have an excellent prognosis, while those with positive LGE are at increased risk, irrespective of LVEF.

Baggiano, A. et al. [6] reviews the new paradigm in the diagnosis of coronary disease by multimodality imaging. In patients with chest pain, coronary CT angiography is emerging as the pivotal technique for first diagnosis. Thereafter, functional techniques should be employed to identify hemodynamically significant stenosis related to patients’ symptoms and future clinical events. In ischemic heart disease, CMR allows the assessment of cardiac structure and function and provides parameters with important prognostic implications, such as left ventricular volumes and LVEF, infarct size, and the presence of microvascular obstruction.

Valente, F. et al. [7], in an interesting article, shows that CMR circumferential strain improves the value of LGE alone for the prediction of functional recovery after STEMI. The improvement of the predictive value was particularly relevant in segments with 50–74% infarct transmurality, where LGE often generates uncertainty and further testing is frequently required to clarify the potential for recovery.

The clinical implementation of speckle-tracking echocardiography has allowed the study of the regional myocardial deformation by global longitudinal strain (GLS) and global circumferential strain (GCS) indices to be more effective in detecting the subclinical alteration of LV function compared to LV EF. The study of Ferrara, F. et al. [8], including 269 healthy subjects, demonstrates that systolic hemodynamic forces are higher in men than women, except for diastolic parameters, and that these are reduced in older age (GLS and LVEF did not change, while GCS increased with age).

Moral, S. et al. [9] reviews the recent advances of multimodality imaging in left atrial assessment. Although TTE is the most widely employed procedure for the calculation of atrial size and function, MDCT and CMR are capable of generating 3D anatomical images. CMR permits the estimation of the amount and location of fibrosis in the atrial wall using late gadolinium enhancement sequences, helping in the treatment of atrial arrhythmias. TEE assists in the evaluation of specific atrial structures, especially in the preparation and performance of invasive treatments. Electroanatomical mapping in the electrophysiology laboratory gives us an electrical view of the left atrium that provides a more accurate treatment of arrhythmias. Thus, although left atrium assessment should be performed in all routine cardiac imaging studies, the use of multimodality is mandatory in order to address the most appropriate treatment in diseases of primarily atrial origin.

In conclusion, the papers collected in this Special Issue highlights the advances made in multimodality imaging and their significant implications in different clinical scenarios.
